# Machine learning models accurately predict clades of proteocephalidean tapeworms (Onchoproteocephalidea) based on host and biogeographical data

**DOI:** 10.1111/cla.12610

**Published:** 2025-03-06

**Authors:** Philippe Vieira Alves, Reinaldo José da Silva, Tomáš Scholz, Alain de Chambrier, José Luis Luque, Anastasiia Duchenko, Daniel Janies, Denis Jacob Machado

**Affiliations:** ^1^ Institute of Biosciences, Department of Biodiversity and Biostatistics, Section of Parasitology São Paulo State University (UNESP) Rua Professor Doutor Antonio Celso Wagner Zanin 250 Botucatu 18618689 Brazil; ^2^ Center for Computational Intelligence to Predict Health and Environmental Risks (CIPHER) University of North Carolina at Charlotte (UNC Charlotte) 9331 Robert D. Snyder Rd Charlotte 28223 NC USA; ^3^ Institute of Parasitology Biology Centre of the Czech Academy of Sciences Branišovská 31 České Budějovice 370 05 Czech Republic; ^4^ Department of Invertebrates Natural History Museum CH‐1211 Geneva 6 PO Box 6434 Switzerland; ^5^ Department of Animal Parasitology Federal Rural University of Rio de Janeiro (UFRRJ) Rod. BR 465, km 7 Seropédica 23890‐000 RJ Brazil; ^6^ Department of Bioinformatics and Genomics, College of Computing and Informatics University of North Carolina at Charlotte (UNC Charlotte) 9331 Robert D. Snyder Rd Charlotte 28223 NC USA

## Abstract

Proteocephalids are a cosmopolitan and diverse group of tapeworms (Cestoda) that have colonized vertebrate hosts in freshwater and terrestrial environments. Despite the ubiquity of the group, key macroevolutionary processes that have driven the group's evolution have yet to be identified. Here, we review the phylogenetic relationships of proteocephalid tapeworms using publicly available (671) and newly generated (91) nucleotide sequences of the nuclear RNA28S and the mitochondrial MT‐CO1 for 537 terminals. The main tree search was carried out under the parsimony optimality criterion, analysing different gene alignments simultaneously. Interestingly, we were not able to recover monophyly of the Proteocephalidae. Additionally, it was difficult to reconcile the tree with host and biogeographical data using traditional character optimization strategies in two dimensions. Therefore, we investigated if host and biogeographical data can be correlated with the parasite clades in a multidimensional space–thus considering multiple layers of information simultaneously. To that end, we used random forests (a class of machine learning models) to test the predictive potential of combined (not individual) host and biogeographical data in the context of the proteocephalid tree. Our resulting models can correctly place 88.85% (on average) of the terminals into eight representative clades. Moreover, we interactively increased the levels of clade perturbation probability and confirmed the expectation that model accuracy negatively correlates with the degree of clade perturbation. Our results show that host and biogeographical data can accurately predict proteocephalid clades in multidimensional space, even though they are difficult to optimize in the parasite tree. These results agree with the assumption that the evolution of proteocephalids is not independent of host and biogeography, and both may provide external support for our tree.

## Introduction

Proteocephalidean tapeworms (Cestoda) are a cosmopolitan group of cestodes that infect freshwater teleosts, non‐avian reptiles and, to a lesser extent, amphibians (de Chambrier et al., 2015, 2017; Scholz and Kuchta, 2022). *Thaumasioscolex didelphidis* Cañeda‐Guzmán, de Chambrier et Scholz, 2001 is the only proteocephalid known to infect a mammal, the black‐eared opossum *Didelphis marsupialis* L. in Mexico (Cañeda‐Guzmán et al., 2001). Initially classified within their own order, Proteocephalidea, they are now grouped under Onchoproteocephalidea along with hook‐bearing species and *Prosobothrium* spp., all of which are exclusively found in elasmobranch hosts as adults (Caira et al., 2014, 2017). For clarity, we will use here “Onchoproteocephalidea I” sensu de Chambrier et al. (2017) and “proteocephalid(s)” when referring to the former members of the Proteocephalidea.

The evolutionary history of proteocephalids remains obscure owing to either the lack of phylogenetic resolution in the tree reconstructions or the absence of any obvious patterns regarding historical processes that have driven the group's evolution (de Chambrier et al., 2015, 2017). The most comprehensive phylogenetic reconstruction for the proteocephalid cestodes, based on the large nuclear ribosomal RNA subunit (RNA28S), did not indicate any clear cospeciation nor zoogeographical patterns, except for some smaller groups such as the clade composed of morphologically diverse species from African catfishes (Siluriformes) (de Chambrier et al., 2015). Instead, the authors indicated that multiple events of host‐switching have occurred throughout the lineages, further suggesting that catfishes have played a key role in the evolution of the group, notably pimelodid catfishes (Pimelodidae) from the Neotropics. Evidence of this intricate evolution is found in the so‐called “Neotropical fish superclade”. Besides fish proteocephalids from South America, such a “superclade” also is invoked for parasites of the Palaearctic and Nearctic regions hosted by salamanders, frogs, turtles, snakes and the bowfin *Amia calva* L. (see de Chambrier et al., 2015; Scholz et al., 2022).

Assuming that parasite phylogenies are influenced by host evolution and environmental factors, machine learning (ML) applications such as random forests can be trained to test whether metadata composed of host taxonomy and biogeographical attributes, including zoogeographical regions, can be reconciled with the parasite phylogenies. Although the complex evolutionary history of proteocephalids hinders the recovery of meaningful patterns from host and zoogeographical data using classical character optimization, random forest algorithms offer promising alternatives by analysing several features simultaneously in a multidimensional space (Boulesteix et al., 2012). If the metadata can be reconciled with the proteocephalid phylogeny, it should be possible to predict the clades of each terminal based on the given metadata.

According to this reasoning, a total lack of congruence between the proteocephalid tree and the host and biogeographical data suggests that the tree does not accurately reflect the evolutionary history of its taxa. Conversely, aligning the proteocephalid tree with host and biogeographical evidence provides independent validation, indicating that the tree accurately represents the evolutionary history of the group.

In this paper, we review the evolutionary relationships of proteocephalid cestodes using publicly available and newly generated sequences of the nuclear RNA28S and the mitochondrially encoded cytochrome c oxidase I (MT‐CO1) genes. Our goals are two‐fold. First, we determine the phylogenetic affinities of the newly sequenced proteocephalids and identify reciprocal monophyletic groups for the clade prediction analysis using a concatenated dataset (RNA28S + MT‐CO1). Second, we investigate whether host and biogeographical attributes, which individually provide limited phylogenetic information, can collectively predict clades using ML approaches. We anticipate that if our tree informs the phylogeny of proteocephalids, we should be able to correlate its clades with host and biogeographical data even if, individually, these features are not phylogenetically informative.

## Methods

### Biological samples and metadata

As part of a long‐term survey on the diversity of fish helminths in the Neotropics, proteocephalid tapeworms were collected from the intestines of catfishes (Siluriformes; 15 spp. in four families), cichlids (Cichlidae; two spp.), characiforms (Anostomidae; two spp.) and the Argentinian silverside *Odontesthes bonariensis* (Valenciennes) (Atherinopidae). Fishes were caught by artisanal fishermen in multiple South American hydrological drainages between 2013 and 2023, mostly in Brazilian rivers. Information about host individuals and their tapeworms, as well as details on sampling localities, can be found in Appendix S1a.

Tapeworms were removed from the host's intestines, placed in saline (0.9% sodium chloride solution) and gently cleaned of the intestinal content. Posterior‐most proglottids were excised and fixed in 96% molecular grade ethanol for molecular analyses (Chervy, 2024). Whenever possible, the remaining strobila and scolex were prepared as hologenophores (sensu Pleijel et al., 2008) as follows. The hologenophores were placed in a small amount of saline and immediately fixed by pouring hot (almost boiling) 4% formaldehyde solution to keep the worms straight. After 2 weeks, the hologenophores were transferred to 70% ethanol before further processing. Given the small body size of *Proteocephalus microscopicus* Woodland, 1935, a subset of entire individuals were fixed in ethanol, whereas paragenophores sensu Pleijel et al. (2008) were fixed using the above‐mentioned approach. These hologenophores were stained with Mayer's carmine, dehydrated in an ethanol series, clarified by eugenol (clove oil), and mounted in Canada balsam as permanent preparations. A subset of all vouchers was not identified to the species level because they were incomplete or immature.

Newly collected specimens are deposited in the Helminthological Collection of the Biosciences Institute (CHIBB), UNESP, Botucatu, São Paulo, Brazil. The following abbreviations for the hydrological drainages are used: AMA, Amazon River basin; PAR, Paraná River basin; TAR, Tocantins‐Araguaia River basin.

### DNA extraction and PCR protocols

Genomic DNA of 58 specimens (see Appendix S1a) was extracted using a DNeasy DNA Blood & Tissue kit (cat. no. 69506; Qiagen, Hilden, Germany) following the manufacturer's instructions. To expand our dataset, a fragment of previously deposited specimens of *Chambriella megacephala* (Woodland, 1934) (Natural History Museum, Geneva, Switzerland, MHNG‐PLAT‐0091863), *Frezella vaucheri* Alves, de Chambrier, Scholz and Luque, 2015 (Helminthological Collection of the Instituto Oswaldo Cruz, Rio de Janeiro, Brazil, CHIOC‐37979a), *Goezeella siluri* Fuhrmann, 1916 (MHNG‐PLAT‐0085161), *Synbranchiella mabelae* Arredondo, Alves and Gil de Pertierra, 2017 (Collection of the Museo Argentino de Ciencias Naturales “Bernardino Rivadavia,” Buenos Aires, Argentina, MACN‐Pa 619/2) and *Proteocephalus sophiae* de Chambrier and Rego, 1994 (MHNG‐PLAT‐0068968, MHNG‐PLAT‐0079847) also were subjected to DNA extraction and downstream analyses.

Polymerase chain reaction amplification of partial RNA28S (D1–D3 domains) and complete MT‐CO1 was performed following the protocol of Alves et al. (2021a). Gel‐checked PCR products were purified using Exonuclease I and FastAP alkaline phosphatase enzymes (cat. nos EN0582, EF0651; Thermo Fisher Scientific, Waltham, MA, USA), and Sanger‐sequenced at SeqMe (Czech Republic) or BPI Biotecnologia (Brazil). PCR primers and internal primers were used for sequencing as in Alves et al. (2021a). Contiguous sequences were assembled and inspected for errors using Geneious Prime 2023.2.1 (www.geneious.com). MT‐CO1 assemblies were trimmed to the protein‐coding region using the echinoderm and flatworm mitochondrial code (translation table 9). Consensus sequences were submitted to NCBI's GenBank database (accession numbers are included in Appendix S1b). Access to genetic data of specimens collected in Brazil was granted by the Brazilian Ministry of Environment (SisGen AE22EAE).

### Sequence alignment and phylogenetics

Sequences generated *de novo* (91) were assembled into two alignments based on the RNA28S and MT‐CO1 datasets, together with selected onchoproteocephalids (671 publicly available sequences). The alignments were performed using the ‐‐auto command of MAFFT v7.490 (Katoh et al., 2002; Katoh and Standley, 2013) implemented in Geneious. This option searches for the best algorithm according to the size of the dataset. Ragged ends in the alignments were either coded as “N” or “?”. The individual alignments for RNA28S (510 terminals, 1942 bp long; Appendix S3a) + MT‐CO1 (253 terminals, 1617 bp long; Appendix S3b) were concatenated in a final matrix containing 537 terminals, 3559 bp long (Appendix S3c). Given that our analyses aim to maximize the explanatory power of the available evidence through a total evidence framework (Kluge, 1989, 2004; Nixon and Carpenter, 1996), terminals containing data from only one of the molecular markers (313 of 537) also were considered in the phylogenetic analyses.

As for the outgroup selection, we leveraged the prior assumption regarding the monophyly of proteocephalids (Caira et al., 2014; Caira and Jensen, 2014; de Chambrier et al., 2015, 2017) and selected representatives of elasmobranch‐hosted onchoproteocephalids (‘Onchoproteocephalidea II’ sensu Caira et al., 2017). Specifically, we included species of *Acanthobothrium* Blanchard, 1848, *Potamotrygonocestus* Brooks and Thorson, 1976, and *Matticestus* Caira, Jensen and Fyler, 2018. Despite our initial assumption, the monophyly of proteocephalids was not supported in a preliminary tree reconstruction (not shown). Therefore, we expanded the analysis to encompass the more distantly related *Pachybothrium hutsoni* (Southwell, 1911), “Tetraphyllidea” relics, and *Clistobothrium montaukensis* Ruhnke, 1993, Phyllobothriidea, based on the tree topology presented by Waeschenbach et al. (2012). The final dataset included 85 outgroup and 452 ingroup taxa, rooted on *C*. *montaukensis*. This large outgroup sampling has three purposes: inferring the order of character transformation, recovering the phylogenetic relationships of ingroup terminals and testing the ingroup monophyly, which is one of the fundamental premises in phylogenetic analyses (Grant, 2019). The GenBank accession numbers and associated metadata of all sequences are provided in Appendix S1b.

In this paper, we focus our results and discussion on a phylogenetic reconstruction experiment performed under the parsimony optimality criterion, analysing all available data simultaneously and applying equal weights to the character state transformations. This choice is based on our interpretation of phylogenetics, which aims to minimize the number of character transformations required to explain the observations. Under this interpretation, unweighted (equally weighted) parsimony analysis is justified, as it reduces the number of hypothesized transformations globally.

Another reason for focusing our analyses using parsimony as a preferred method over maximum‐likelihood or Bayesian inference is our concern for the treatment of insertion and deletion events (“Indels” or gaps, represented by “‐” in the sequence alignment) as evolutionary events that are distinct from missing or inapplicable characters (represented by “?” or “N”). Unlike model‐based approaches such as standard maximum‐likelihood analysis, which treat gaps (indels) as missing data, parsimony may consider the historical events of insertions and deletions as a fifth character state (see Jacob Machado et al., 2021, and references therein). Our dataset is already missing‐data rich (*c*. 42%), so the parsimony analysis conducted here aims to leverage this evidence as much as possible to maximize the explanatory power of the concatenated RNA28S + MT‐CO1 matrix.

Tree searches under the parsimony criterion were performed in TNT v.1.6 (Goloboff et al., 2008) using its new technologies search (Nixon, 1999; Goloboff et al., 2008). Five individual runs (Appendix S3d) were executed using multiple replications of combined sectorial searches, drifting, ratchet and fusing, as implemented by the TNT command “xmult= level 5 rep 1000.” Following this exhaustive exploration of tree space, a strict consensus tree was generated using the most parsimonious trees (Appendix S3e) of previous analyses using the command “nelsen” (Appendix S3f). The branch length also was calculated (Appendix S3g). Last, the branch supports and clade frequencies were estimated by calculating Goodman–Bremer (GB) and jackknife values, respectively (Appendix S3h).

A separate maximum‐likelihood analysis was performed for the interested reader and is described in Appendix S5. In short, we utilized IQ‐TREE v.2.3.4 COVID‐edition, a multicore‐enabled software for phylogenetic inference (Nguyen et al., 2015), to conduct a maximum‐likelihood analysis with partitioned data. The command used the ‐p partitions.nexus flag to specify a partitioned dataset in NEXUS format, enabling the application of different evolutionary models to distinct data partitions as selected in ModelFinder (Kalyaanamoorthy et al., 2017). The search strategy was controlled by the ‐ninit 100 ‐nstop 100 parameters, indicating that 100 initial trees were generated, and the search was stopped after 100 unsuccessful topology improvements. The root was designated as *C*. *montaukensis* using the ‐o flag. Parallel computation was enabled with ‐T 6, leveraging six CPU threads to enhance computational efficiency. The –runs 10 option directed the program to conduct ten independent ML tree searches to assess convergence. Statistical support for inferred phylogenies was evaluated using 1000 replicates of both ultrafast bootstrap (UFBoot) with ‐B 1000 (Minh et al., 2013) and approximate likelihood‐ratio tests (SH‐aLRT) with –alrt 1000 (Guindon et al., 2010), providing branch support measures.

We used YBYRÁ (Jacob Machado, 2015) to compare the topologies obtained with parsimony and maximum‐likelihood, looking for shared and unique clades between both trees. This analysis and its results are described in Appendix S5.

### Character optimization

We used YBYRÁ (Jacob Machado, 2015; script “ybyra_apo.py”) to test whether individual host and zoogeographical attributes can be used to inform clades recovered in our molecular‐based proteocephalid tree. One of the most parsimonious trees was used to map the character (host and biogeographical) transformation events in the internal nodes of clades with three or more terminal nodes and categorize nonambiguous transformations as: synapomorphic with derived states that are exclusive to the clade (unique); homoplastic and with derived states that are exclusive to the clade (private); or homoplastic and with derived states that are not exclusive to the clade (nonprivate). (Jacob Machado, 2015).

The following categorical attributes (characters) were considered: CatE1, habitat; CatE2, aquatic ecosystems; CatHC, host class; CatHO, host order; CatL1, zoogeographical region (according to Holt et al., 2013); and CatL2, continent. Data from paratenic hosts were coded as nonapplicable (“NA”) to avoid introducing noise in the analysis. Detailed information about the characters and their states, as well as how they were coded, is available in Appendices S1c and S1d; see also Appendix S2 files.

### Machine learning analysis

The analysis was performed using a class of supervised machine‐learning models called random forests. Random forests are a nonparametric method that can predict a response variable based on a potentially large number of predictor variables (Rothacher and Strobl, 2023). Random forests were originally introduced by Breiman (2001) and are known for their high accuracy, reduced susceptibility to overfitting and ability to handle high‐dimensional data (Burkov, 2019).

Random forests allow easy integration of data from multiple sources while combining the benefits of interpretability and flexibility (McAlexander and Mentch, 2020). Moreover, different from the characters in a phylogenetic matrix, these variables can be dependent and hierarchical (e.g. order, family, genera and species) and do not necessarily indicate homology hypotheses.

Metadata were pre‐processed and organized using Python v.3.8.10 scripts with the Pandas v.1.5.3 and Numpy v.1.24.4 libraries. Columns or rows containing missing data were removed before analysis. The curated host and biogeographical dataset included ten features for 494 terminals (*c*. 8% of taxa excluded). Data from intermediate or paratenic hosts were not considered. The following categorical attributes were used: CatHC–host class; CatHO, host order; CatHF, host family; CatHG, host genus; CatHS, host species; CatE1, habitat; CatE2, aquatic ecosystems; CatL1, zoogeographical region; CatL2, continent; and CatL5–country (for details, see Appendix S1e).

The eight clades selected from the proteocephalid tree were labelled by colour (see Appendix S1f). Then, these files were used as input for the analysis. The final table contained 4940 data points. Host information comprised five classes, 28 orders, 63 families, 116 genera and 170 species of hosts. Biogeographical data included two types of environment (aquatic or terrestrial) and three types of aquatic environments based on salinity (freshwater, brackish and saltwater). It also included ten zoogeographical regions (according to Holt et al., 2013), seven continents, and 42 countries or river basins.

The random forest classifier was coded in Python v.3.8.10 using Scikit‐learn v.1.3.2. Each random forest experiment comprised 100 decision trees (Appendix S4a). We transformed the categorical values into vectorized, numerical binary features using one‐hot encoding, preserving information without ranking it (Burkov, 2019). Labels were modified with 0% to 100% chance of perturbation at 1% increments. Each perturbation level corresponds to the chance of assigning a terminal to a random label (clade). After that, and for each perturbation level, 75% of the data were randomly selected for training and the remaining 25% were used for validation. We conducted ten replicates per perturbation level.

**Fig. 1:**
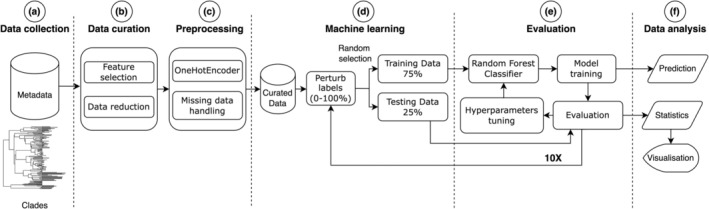
Flowchart summarizing individual steps of the ML analysis. (a) Data collection: host taxonomy and biogeographical metadata were compiled, whereas clades were selected from the proteocephalid phylogeny. (b) Data curation: metadata were manually curated, including removal of rows related to intermediate or paratenic hosts. (c) Preprocessing: qualitative data were processed to handle missing data and transform categorical values with one‐hot encoding. (d) Machine learning training: labels were modified with 0% to 100% chance of perturbation at 1% increments; 75% of data, for each perturbation level, were randomly selected to train the random forest classifier. The remaining 25% of data were used to measure accuracy and calculate importance values. (e) Model evaluation and replication: training and evaluation procedures were iteratively repeated, and hyperparameter tuning was conducted. Random data selection, training and evaluation were repeated ten times per perturbation level. (f) Data analysis: generation of tables and graphs by the trained models.

For the perturbation test, we calculated a linear regression model and retrieved the corresponding $R^{2}$ and *p*‐value using Scipy v.1.10.1 and Statsmodels v.0.14.1. We used Matplotlib v.3.7.4 and Seaborn v.0.11.2 to visualize the results. Individual steps of ML analysis are described in Fig. 1; additional files are in Appendix S4, including the input data required to run the analysis and the results generated.

We also trained a separate ML model based on the clades obtained in the maximum‐likelihood analysis and otherwise following the same procedures described above. This analysis and its results are described in Appendix S5.

## Results

### A revised molecular phylogeny of proteocephalids

In total, three equally parsimonious trees were found for the concatenated dataset (RNA28S + MT‐CO1), each with 26 527 steps. Acknowledging the susceptibility of parsimony analysis to long‐branch attraction (LBA) (Bergsten, 2005), we screened the entire tree for two long branches that, although not true sisters, appeared as such within clades composed of short branches. We found none. Selected clades are briefly characterized below. Files related to the tree search, including the best heuristic results and the strict consensus with relative Goodman–Bremer and Jackknife values, are provided in Appendices S3d–S3k.

**Fig. 2:**
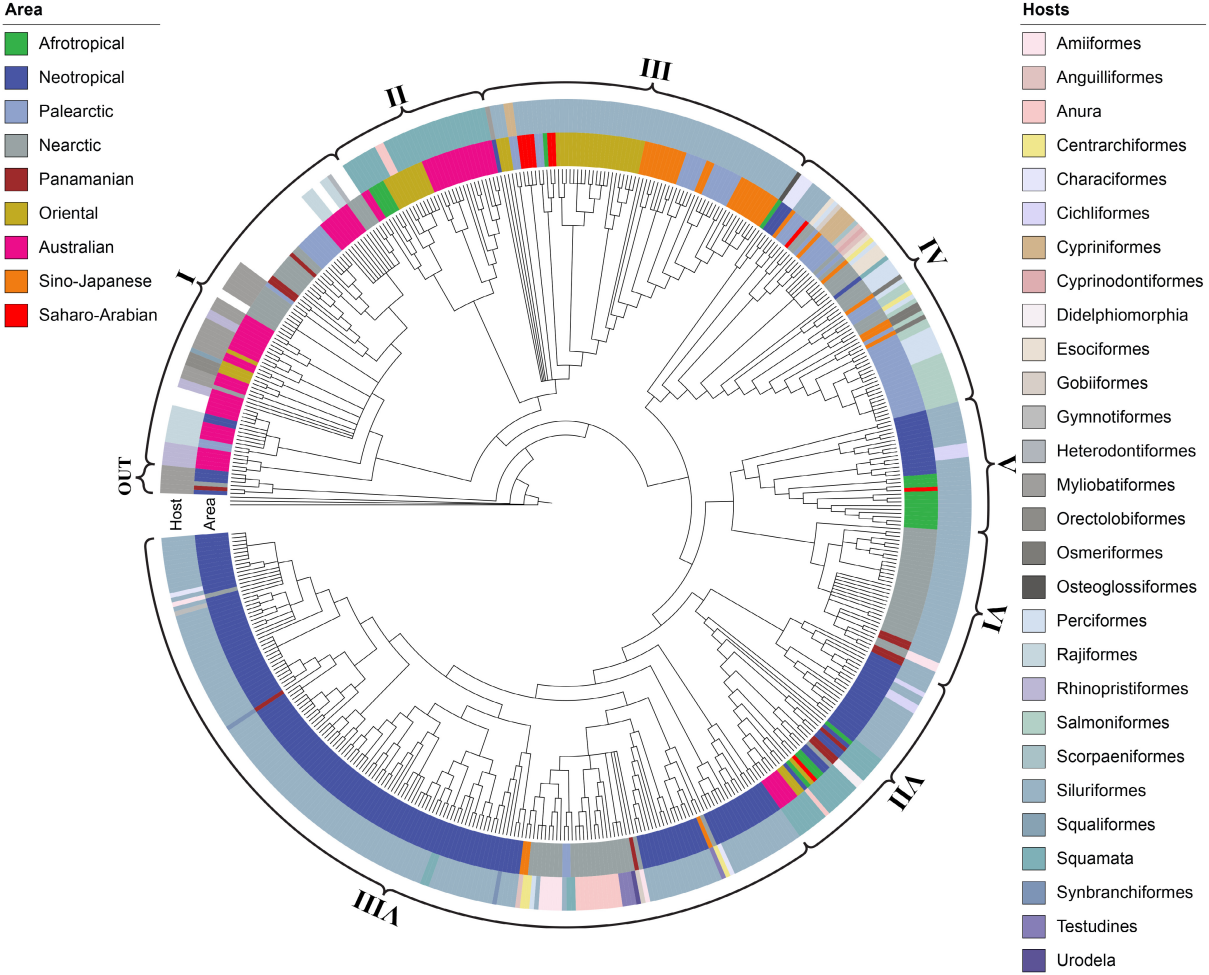
Circular strict consensus tree inferred from the concatenated RNA28S + MT‐CO1 matrix under the parsimony criterion. Clades defined in curly braces as “I–VIII” represent the eight selected clades for prediction. The outer and the inner coloured circles refer to the host orders and zoogeographical regions, respectively. Note that none of the clades can be easily characterized by the mapped attributes. The circular tree and annotations were generated in the iTOL web server (www.itol.embl.de).

**Fig. 3:**
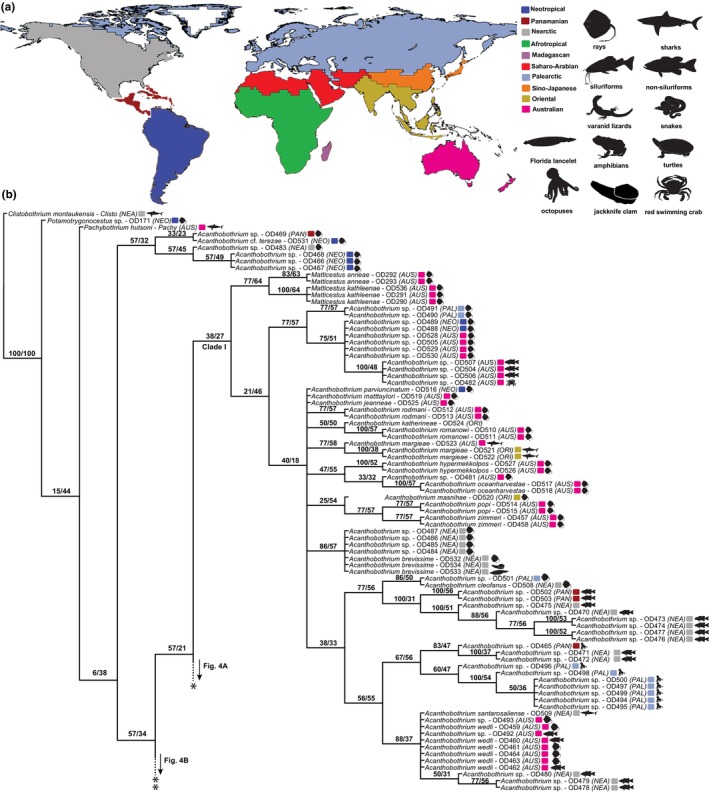
Zoogeographical regions (according to Holt et al., 2013) and host silhouettes (www.phylopic.org/) mapped onto the phylogenetic tree (a). Strict consensus tree as inferred from the concatenated RNA28S + MT‐CO1 matrix under the parsimony criterion, part 1 of 7; clade I outlined (b). Relative Goodman–Bremer supports/jackknife clade frequencies are displayed at the top of each branch. The asterisks indicate the branch that continues in Fig. 4. AUS, Australian; NEA, Nearctic; NEO, Neotropical; PAL, Palaearctic; PAN, Panamanian; ORI, Oriental.

**Fig. 4:**
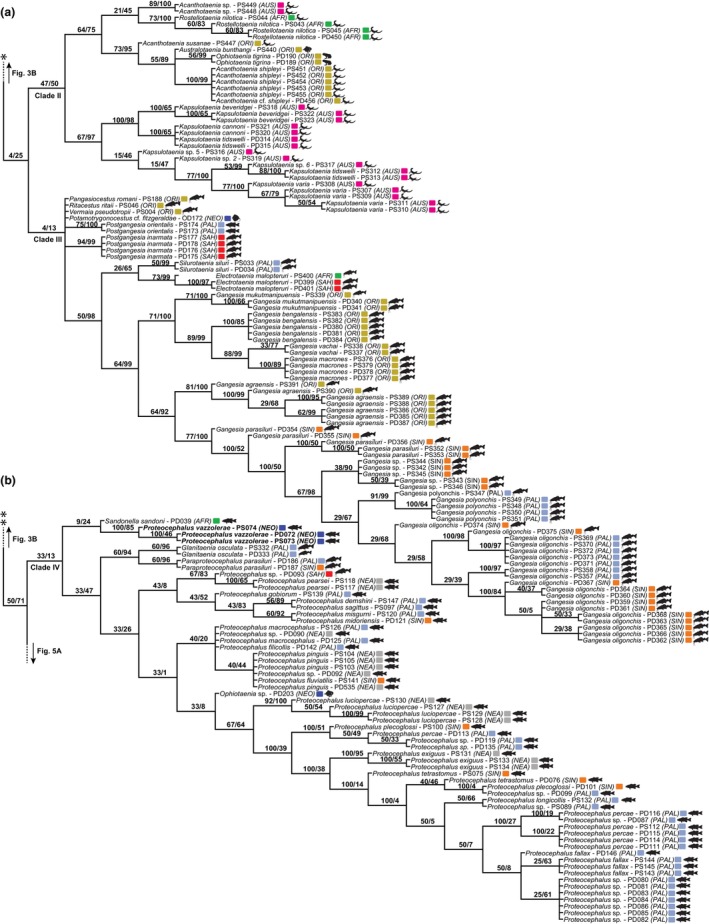
Strict consensus tree from parsimony analysis, part 2 of 7 (clades II and III outlined) (a) and part 3 of 7 (clade IV outlined) (b). Relative Goodman–Bremer supports/jackknife clade frequencies are displayed at the top of each branch. The asterisks indicate the branch that continues in Fig. 3. New sequences are in bold. AFR, Afrotropical; AUS, Australian; NEA, Nearctic; NEO, Neotropical; PAL, Palaearctic; ORI, Oriental; SAH, Saharo‐Arabian; SIN, Sino‐Japanese.

**Fig. 5:**
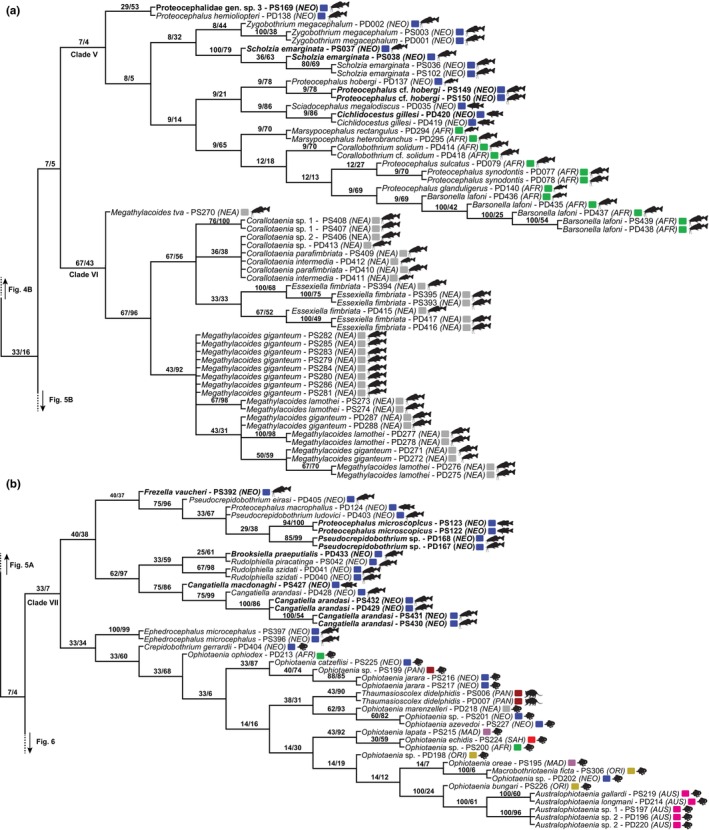
Strict consensus tree from parsimony analysis, part 4 of 7 (clades V and VI outlined) (a) and part 5 of 7 (clade VII outlined) (b). Relative Goodman‐Bremer supports/Jackknife clade frequencies are displayed at the top of each branch. New sequences are in bold. AFR, Afrotropical; AUS, Australian; NEA, Nearctic; NEO, Neotropical; PAN, Panamanian; MAD, Madagascan; ORI, Oriental; SAH, Saharo‐Arabian.

**Fig. 6:**
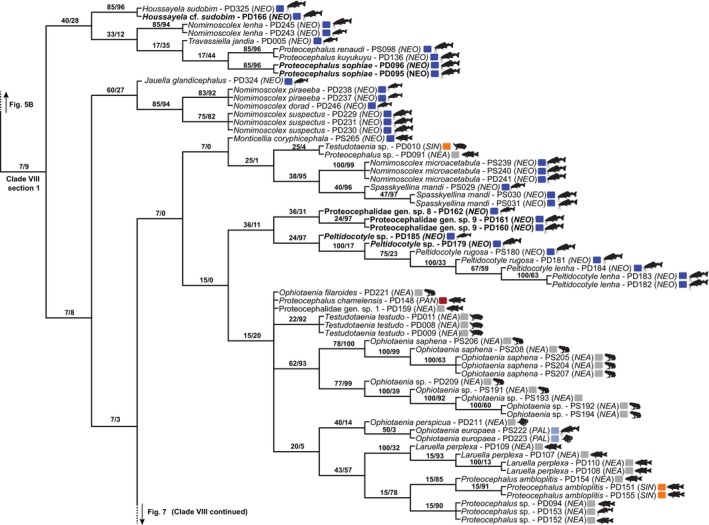
Strict consensus tree from parsimony analysis, part 6 of 7 (clade VIII, section 1, outlined). Relative Goodman–Bremer supports/Jackknife clade frequencies are displayed at the top of each branch. New sequences are in bold. NEA, Nearctic; NEO, Neotropical; PAN, Panamanian; PAL, Palaearctic; SIN, Sino‐Japanese.

**Fig. 7:**
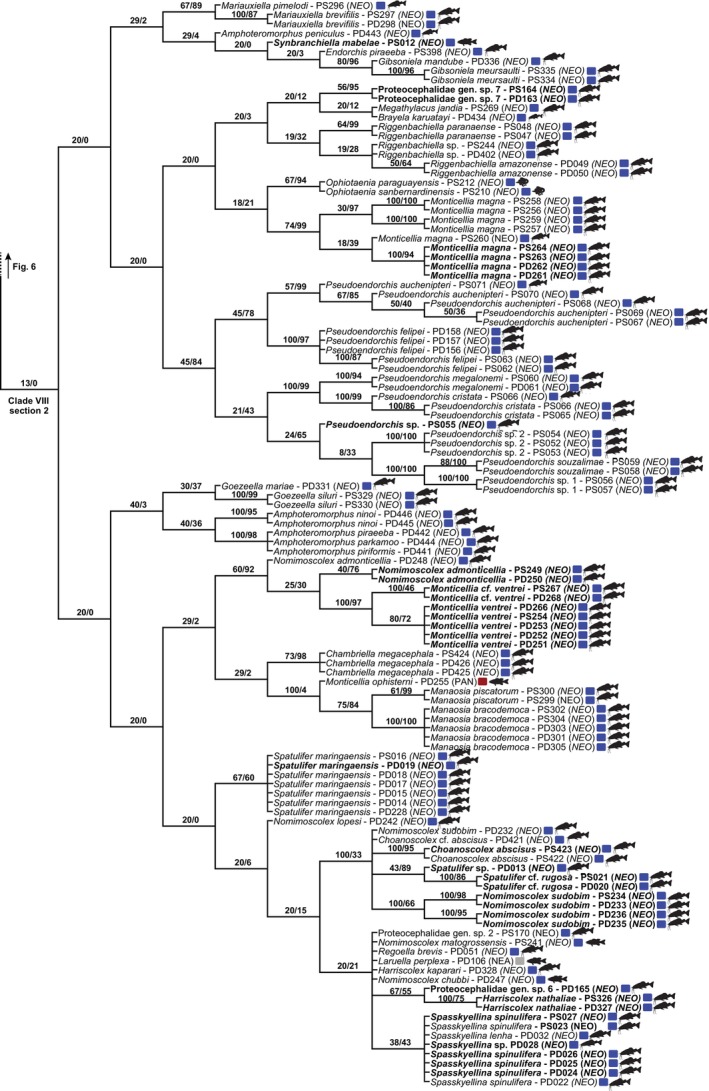
Strict consensus tree from parsimony analysis, part 7 of 7 (clade VIII, section 2, outlined). Relative Goodman–Bremer supports/Jackknife clade frequencies are displayed at the top of each branch. New sequences are in bold. NEA, Nearctic; NEO, Neotropical; PAN, Panamanian.

Eight reciprocally monophyletic Clades (I–VIII), including >25 terminals each, were identified in the strict consensus tree (Fig. 2). These clades were used to train the random forest models. This tree was divided by the selected clades to allow better topology visualization (Figs 3, 4, 5, 6, 7).

Clade I is formed by the outgroup representatives belonging to the elasmobranch‐hosted onchoproteocephalid genera *Matticestus* and *Acanthobothrium* (Fig. 3b). It was one of the selected clades because the monophyly of our ingroup is not recovered with the inclusion of these terminals as Clades II + III are more closely related to this group than to the remaining proteocephalids. The sequences of *Acanthobothrium* spp. and *Potamotrygonocestus* sp. that did not cluster in this clade were only represented by MT‐COI data, whereas the remaining members of Clade I were only represented by RNA28S data.

Clade II is formed by members of the Acanthotaeniinae, parasites of non‐avian reptiles and amphibians of the Afrotropical, Oriental and Australian realms, as well as *Ophiotaenia tigrina* (Woodland, 1925) (Fig. 4a). This latter belongs to the polyphyletic *Ophiotaenia* La Rue, 1911 (former Proteocephalinae), the type species of which, *O*. *perspicua* La Rue, 1911, is positioned elsewhere in the tree. Among the acanthotaeniine proteocephalids, only *Acanthotaenia* von Linstow, 1903 was found as nonmonophyletic.

Sister to Clade II, Clade III consists mainly of Gangesiinae, primarily from catfishes (Siluriformes) in the Old World (Fig. 4a). All seven genera assigned to this subfamily are sampled in our tree, five of which are monotypic. The species‐rich *Gangesia* Woodland, 1924 is monophyletic, yet the two species of *Postgangesia* Akhmerov, 1969 appeared in a polytomy so its monophyly could not be confirmed. The monophyly of the Gangesiinae is disrupted by the inclusion of *Potamotrygonocestus* cf. *fitzgeraldae* Marques, Brooks and Araújo, 2003 ex *Potamotrygon tatianae* Silva and Carvalho from the Peruvian Amazon.

The majority of terminals in Clade IV belongs to the monophyletic *Proteocephalus*‐aggregate sensu de Chambrier et al. (2004), a well‐known group of proteocephalids in freshwater teleosts of Holarctic distribution (Fig. 4b). The presence of *Ophiotaenia* sp. (PD203; KT375455) in this clade is likely to be an artefact as >95% of this sequence is composed of missing data in our matrix.

The *Proteocephalus*‐aggregate is sister to *Glanitaenia osculata* (Goeze, 1782) and *Paraproteocephalus parasiluri* (Zmeev, 1936) both found in silurid catfish (Siluridae) from the Palaearctic region. Representing the earliest diverging taxa in Clade IV are the enigmatic *Sandonella sandoni* (Lynsdale, 1960) from the osteoglossiform fish *Heterotis niloticus* (Cuvier) (Arapaimidae) in the Afrotropical region, and *Proteocephalus vazzolerae* Pavanelli and Takemoto, 1995 from anostomid fishes (Characiformes, Anostomidae) of the Upper PAR, Neotropics.

Clade V is the smallest of the selected clades and is composed of a monophyletic assemblage of morphologically distinct proteocephalids in catfishes from the Afrotropical region, as well as a heterogeneous group parasitic in large catfish and cichlid hosts of the Neotropical region (Fig. 5a).

Previously assigned to the polyphyletic Corallobothriinae, representatives of Clade VI (Fig. 5a) are now included in their own subfamily, Essexiellinae, and are exclusively found in ictalurid catfishes (Ictaluridae) of the Nearctic region (Scholz et al., 2020). Although sequences of the monotypic *Essexiella* Scholz, de Chambrier, Mariaux and Kuchta, 2011 clustered together in a single internal clade, the monophyly of *Corallotaenia* spp. was not confirmed and that of *Megathylacoides* spp. was rejected.

Clade VII is one of the most eclectic clades in taxon composition and can be described by its two subclades (Fig. 5b). One subclade consists of fish proteocephalids from the Neotropical region and the other primarily consists of cestodes from snakes across different zoogeographical regions. The latter clade also includes the morphologically unique *T*. *didelphidis* and *Ephedrocephalus microcephalus* Diesing, 1850, parasites of a New World mammal and fish, respectively.

The last and largest selected proteocephalid group, Clade VIII, is predominantly formed of fish cestodes from the Neotropical region, with catfishes of the family Pimelodidae as the main host group (Figs 6, 7). However, cestodes of amphibians, non‐avian reptiles, and fishes other than catfishes from different parts of the globe also can be found in this heterogeneous clade, whose evolutionary history is challenging to reconstruct.

### Interrelationships of newly sequenced taxa

Besides the publicly available sequences, our phylogeny included novel data for 30 species/species‐level lineages of fish proteocephalids from the Neotropics. *Proteocephalus vazzolerae* was found to be the earliest diverging Neotropical fish proteocephalid, clustering in Clade IV (Fig. 4b). Representatives from *Leporinus friderici* (Bloch) from the Paranapanema and Paraná Rivers (both PAR) are sister to the single specimen from another anostomid, *Schizodon borellii* (Boulenger) (new host), collected in the latter river.

Six novel isolates are among the Neotropical fish proteocephalid taxa in Clade V (Fig. 5a). Sequences of immature specimens tentatively identified as *Pr*. cf. *hobergi* de Chambrier and Vaucher, 1999 ex *Oxydoras niger* (Valenciennes) from the AMA clustered along with a conspecific isolate from *O*. *kneri* Bleeker from the PAR. Also clustering with conspecifics but in different subclades are *Cichlidocestus gillesi* de Chambrier, Pinacho‐Pinacho, Hernández‐Orts and Scholz, 2017 from *Aequidens tetramerus* (Heckel) (new host) in the Marapanim River (northern coastal basin of Brazil; new locality), and *Scholzia emarginata* (Diesing, 1850) from both *Phractocephalus hemioliopterus* (Bloch and Schneider) and *Pseudoplatystoma fasciatum* (L.) (new host) in the TAR (new locality). One unidentified proteocephalid, Proteocephalidae gen. sp. 3 ex *Pseudoplatystoma* cf. *reticulatum* Eigenmann and Eigenmann in the TAR further clustered as sister to *Proteocephalus hemioliopteri* de Chambrier and Vaucher, 1997 ex *Ph*. *hemioliopterus*.

As for Clade VII (Fig. 5b), *Proteocephalus microscopicus* Woodland, 1935 ex *Cichla kelberi* Kullander and Ferreira in the PAR exhibited a sister relationship to *Pseudocrepidobothrium* sp. ex *Ps*. *reticulatum* in the AMA. They are further nested in a clade that also includes the previously sequenced *Pseudocrepidobothrium eirasi* (Rego and de Chambrier, 1995), *Pr*. *macrophallus* (Diesing, 1850) and *Frezella vaucheri* Alves, de Chambrier, Scholz and Luque, 2015. New sequences of *Cangatiella arandasi* Pavanelli and Machado, 1991 ex *Trachelyopterus galeatus* (L.) from the PAR nested within a clade alongside its conspecific from the same host and basin, sister to *C*. *macdonaghi* (Szidat and Nani, 1951) ex *Odonthestes bonariensis* (Valenciennes) from an Argentinian lagoon; this finding supports the monophyly of the genus. The *Cangatiella* clade, in turn, is sister to *Brooksiella praeputialis* (Rego, Santos and Silva, 1974) and *Rudolphiella* spp.

The majority of newly sequenced taxa are nested in the large Clade VIII (Figs 6, 7). Sequences of *Nomimoscolex admonticellia* (Woodland, 1934) from the TAR (new locality) and AMA clustered in a clade together with *Monticellia ventrei* de Chambrier and Vaucher, 1999 from the AMA and PAR, and *M*. cf. *ventrei* from the TAR (new locality). All specimens in this clade are from the widespread pimelodid catfish *Pinirampus pirinampu* (Spix and Agassiz).

The monophyly of the genus *Harriscolex* Rego, 1987 is not supported as first sequences of *Harriscolex nathaliae* Gil de Pertierra and de Chambrier, 2013 ex *Ps*. *corruscans* (Spix and Agassiz) from the PAR did not cluster along with the congeneric and type species *H*. *kaparari* (Woodland, 1935).

Novel data of *Spasskyellina spinulifera* (Woodland, 1935) confirmed that this proteocephalid is widely distributed (AMA, PAR, TAR—new locality) and can be found in different species of the large pimelodid catfish *Pseudoplatystoma* Bleeker.

### Characters that are difficult to optimize

The traditional optimization of host and biogeographical metadata over one of the heuristically parsimonious trees resulted in a highly polytomic cladogram owing to the lack of synapomorphic transformations in most branches (see Appendix S2g). Among the few surviving clades that were supported by synapomorphies, none could be described by unique, nonhomoplastic character transformations. In other words, no unequivocal diagnoses of clades were possible based on host and biogeography alone. However, a few unique sets of otherwise nonunique transformations could be used to support a limited number of clades, such as that formed by cestodes from snakes from distant zoogeographical regions (our Clade VII; see Fig. 5b and Appendix S2g).

### Reconciling parasite phylogeny with host and biogeography

The random forest experiment demonstrates that integrating host and biogeographical metadata with the current proteocephalid tree is feasible and effective. Without perturbation, our model accurately placed 88.85% (average of ten replicates) of the 494 tapeworm terminals in their corresponding clades. This means that, of 100 terminals in our phylogenetic tree, the machine‐learning models can correctly predict the clade affiliation of nearly 89 terminals.

**Fig. 8:**
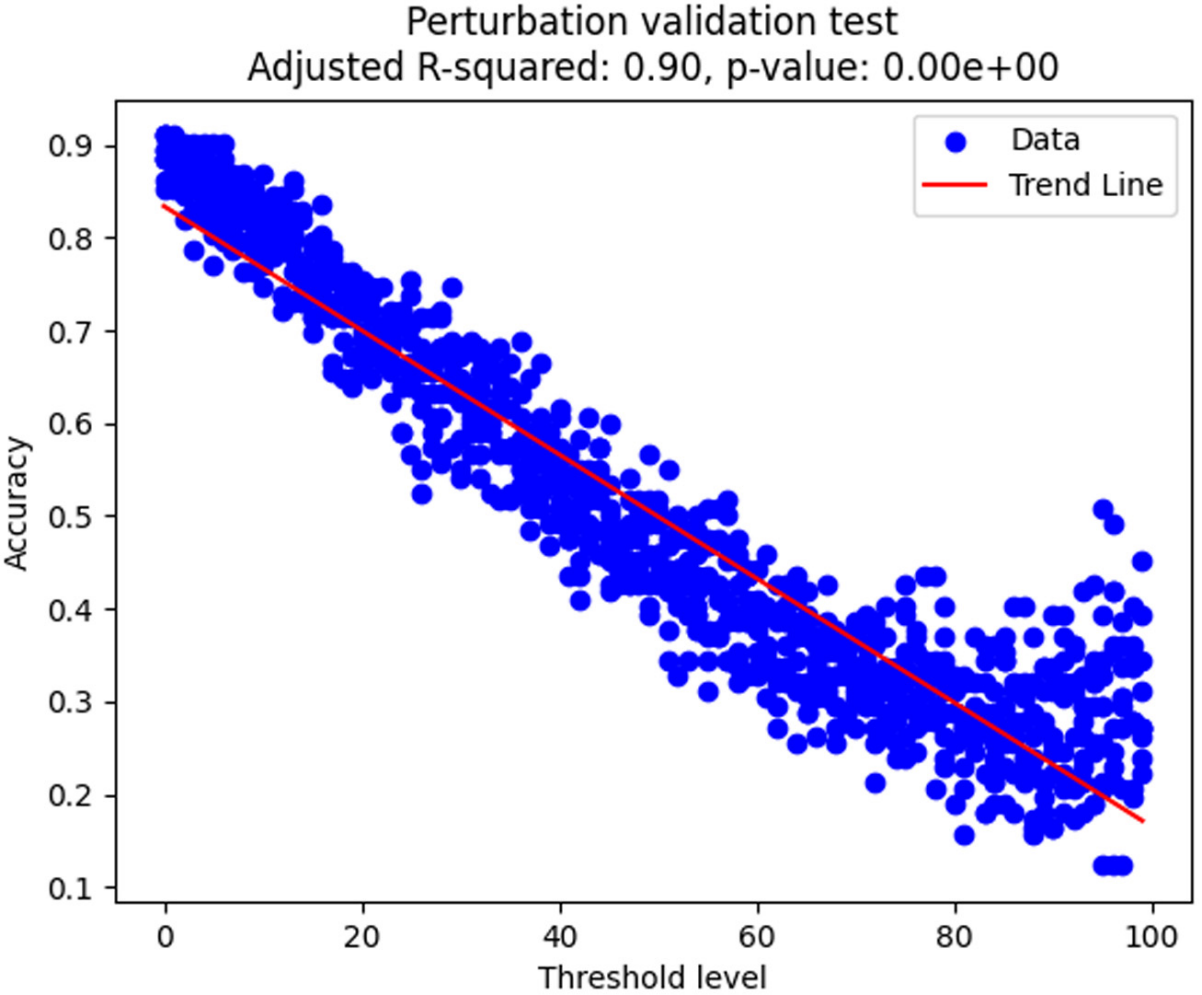
Impact of clade perturbation on the random forest model performance. Accuracy is shown at increasing perturbation levels (1% increments), each with ten replicates.

**Fig. 9:**
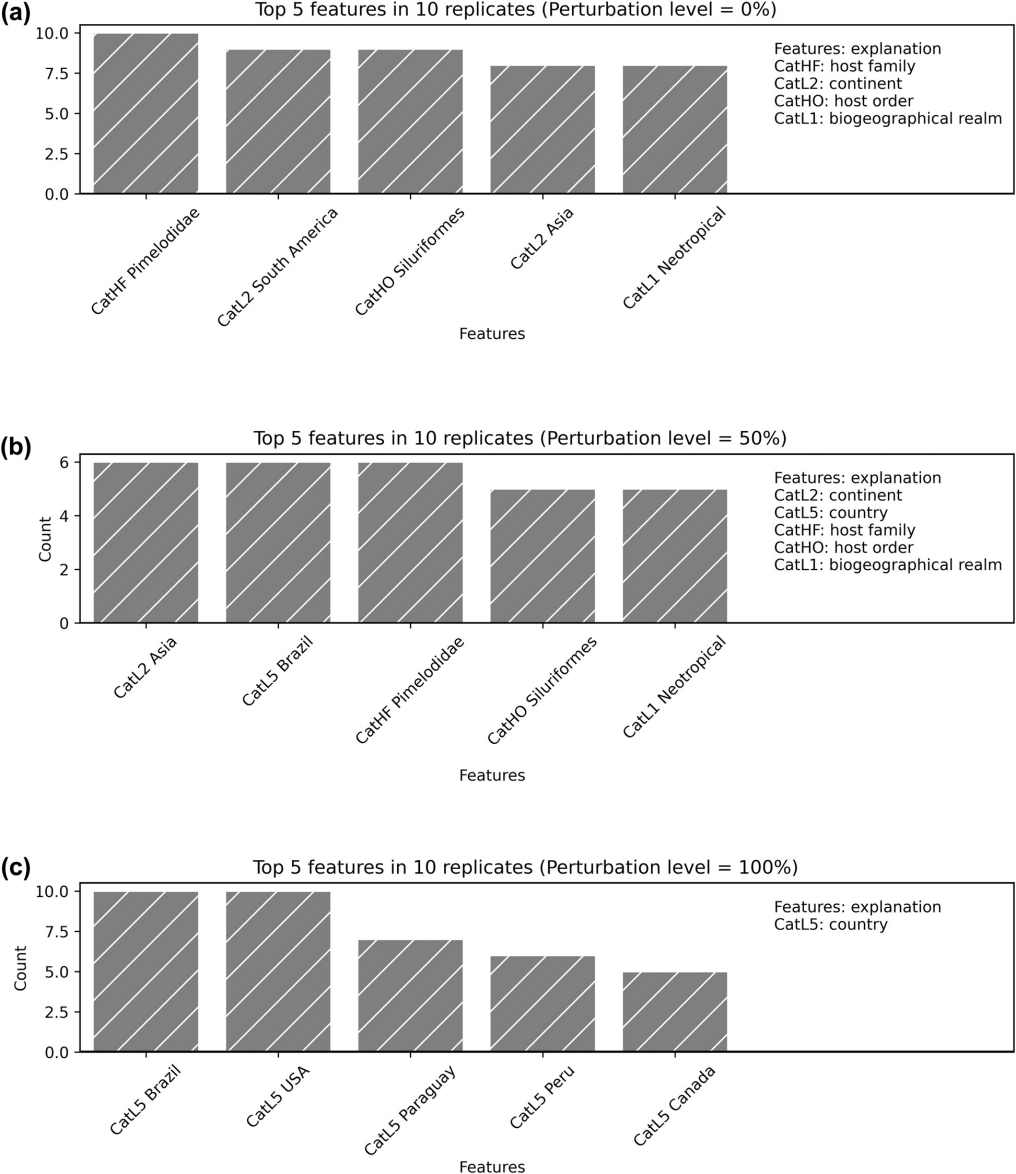
Most important host/biogeographical metadata at increasing levels of clade perturbance, each with ten replicates: perturbation level = 0% (a), 50% (b) and 100% (c). Note that at high perturbation levels, fewer biologically relevant attributes gain importance.

The tree perturbation analysis, which involved randomly assigning terminals to different clades, showed that the model's accuracy negatively correlates with the degree of perturbation ($R^{2}$ = 0.90; Fig. 8). Additionally, the most important features for predicting the proteocephalid clades vary according to the degree of perturbance in the analyses. Although host family and order, continent and zoogeographical realm were relevant without perturbation, countries–including small ones–were the most important at 100% perturbation level (Fig. 9).

### Comparison between the parsimony and the maximum‐likelihood trees

A sensitivity analysis conducted in YBYRÁ revealed that 289 clades (74.1%) were identical between parsimony and maximum‐likelihood trees, whereas 101 clades (25.9%) differed. Given these discrepancies, we selected target clades from the maximum‐likelihood tree and assessed the predictive power of host and biogeographical attributes using the random forest algorithm. Without perturbation, the model's accuracy using clades of the maximum‐likelihood analysis averaged 87.60%, slightly lower than the 88.85% observed for parsimony‐based clades. We provide the maximum‐likelihood tree and associated data in Appendix S5.

## Discussion

The application of molecular taxonomy and phylogenetics has revolutionized our understanding of the interrelationships and species boundaries of proteocephalid tapeworms. However, RNA28S and/or MT‐CO1 molecular phylogenies alone seem insufficient to elucidate the evolution of the group, making it difficult to propose higher‐level rearrangements in the proteocephalid classification schemes (de Chambrier et al., 2015, 2017). With few exceptions, the lack of morphological synapomorphies or any meaningful pattern related to historical processes, such as host association and biogeographical distribution, adds complexity to this challenging task.

Our findings shed more light on the importance of host and biogeographical attributes to proteocephalid evolution, whereas the newly generated sequences pave the way for future attempts to place the classification of proteocephalids in line with phylogenetic information.

### Wildcard terminals and odd relationships

In our attempt to leverage the total molecular evidence available to assess the interrelationships of proteocephalids, we conducted tree searches using a concatenated RNA28S + MT‐COI dataset rich in missing data. We acknowledge that the amount of missing data can contribute to the wildcard behaviour of certain terminals [e.g., *Ophiotaenia* sp. (PD203, Clade IV), *Proteocephalus* sp. (PD090, Clade IV) and *Proteocephalus* sp. (PD091, Clade VIII)]. However, the amount of missing data *per se* does not lead terminals to behave as rogue taxa (see Araujo‐Vieira et al., 2019, and references cited therein). Although the evolutionary affinities of a few terminals, such as the outgroup representatives *Potamotrygonocestus* sp. (OD171) and *Acantobothrium* spp. (OD466–469, OD483), represent an artefact caused by complementary missing data (i.e. one group having only RNA28S and the other only MT‐CO1 sequence; see above and Fig. 3b), most taxa affected by the missing data problem exhibited sister relationships that agree with the topology of single‐gene analyses using a somewhat similar dataset with fewer missing data (compare our tree topology 3, 4, 5, 6, 7 with fig. 1 of de Chambrier et al., 2015). Moreover, the high predictive power of the ML experiment, which did not directly rely on the phylogenetic tree, suggests that our parsimony tree is overall informative and accurate given the available sequences and the likely influence of host and biogeography on the proteocephalid evolution.

Additional odd relationships in our phylogeny deserve attention. The monophyly of Acanthotaeniinae is not supported with the inclusion of *O*. *tigrina* (see Fig. 4a, Clade II). This poorly known species was originally described as *Proteocephalus tigrinus* Woodland, 1925 from the bullfrog *Rana tigrina* (= *Hoplobatrachus tigerinus* [Daudin], Dicroglossidae) in India (Woodland, 1925). Since then, it has been transferred to different genera without critical evaluation of its morphology (de Chambrier et al., 2006).

In our analysis, *O*. *tigrina* (PD189, 190; publicly available sequences lacking associated publications) appeared in a polytomy together with *Acanthotaenia shipleyi* von Linstow, 1903 and *Australotaenia bunthangi* de Chambrier and Scholz, 2012. The latter is the only member of *Australotaenia* de Chambrier and de Chambrier, 2010 that parasitizes snakes and is found outside Australia; the remaining three congeners are found in Australian hylid frogs (de Chambrier and Scholz, 2024). Further morphological and molecular evaluation of *O*. *tigrina* may reveal that this species belongs to *Australotaenia* but it is clearly not a member of *Ophiotaenia* sensu stricto (de Chambrier et al., 2023).

In a similar way to Acanthotaeniinae, Gangesiinae is not monophyletic with the inclusion of *Potamotrygonocestus* cf. *fitzgeraldae* (OD172) found in freshwater stingrays in the Amazonia (see Fig. 4a, Clade III). This RNA28S sequence (KF685773) from a vouchered specimen (LRP‐8288) was published (Caira et al., 2014) and is not among those 10% with a higher amount of missing data; it could be a misidentification case but stingrays have never been reported as harbouring proteocephalids, so the odds are negligible. Caira et al. (2014) and Caira et al. (2018) also recovered this species more closely related to Onchoproteocephalidea I than II sensu Caira et al. (2017), yet with low branch support. A more in‐depth study on the morphology and evolution of this taxon may provide insights into the transition from elasmobranch to bony fish hosts that have occurred in the natural history of the Onchoproteocephalidea.

Another intriguing relationship that caught our attention is the position of *Laruella perplexa* (La Rue, 1911) (PD106) from the bowfin *Amia calva* in Canada. It clustered with Neotropical proteocephalids parasitizing evolutionarily younger fish hosts (Fig. 7, Clade VIII section 2), apart from other isolates of *L*. *perplexa* from bowfin in the Nearctic region (Fig. 6, Clade VIII section 1) (de Chambrier et al., 2009). Recent molecular phylogenies also have suggested that this specimen (PD106; AJ275228), originally assigned to *L*. *perplexa* by de Chambrier et al. (2004), actually belongs to a different and likely undescribed species (Scholz et al., 2024).

### Insights from the newly sequenced taxa

The newly sequenced material primarily comprises proteocephalids from South America's three largest hydrological drainages, including the first molecular data from cestodes of the poorly studied TAR. Our findings revealed putative new species and new host associations, indicating a more relaxed host specificity for some taxa. Additionally, the results expanded the known zoogeographical distribution of several species (see Appendix S1a) and prompted concerns regarding the taxonomic status of certain taxa.

The unidentified proteocephalids, labelled as Proteocephalidae gen. sp., represent putative new species that should be formally described based on freshly collected, complete, and fully mature specimens. Additionally, *Peltidocotyle* sp. (PD185, PD179), *Pseudocrepidobothrium* sp. (PD167, PD168), *Pseudoendorchis* sp. (PS055) and *Spatulifer* sp. (PD013) bear the typical scolex morphology associated with their respective genera and are likely to represent undescribed species awaiting formal description.

The close relationship between *Pseudocrepidobotrium* spp., *Pr*. *macrophallus* and *Pr*. *microscopicus* (Clade VII) also is supported by ultrastructural features. Scanning electron microscopical (SEM) observations (unpublished data) have revealed that some specimens of the latter two species also possess a scolex with four uniloculate, posteriorly notched suckers (inverted heart‐shaped suckers) (Ruedi and de Chambrier, 2012; Arredondo et al., 2014), a feature used to diagnose *Pseudocrepidobothrium* spp. These species also are among the smallest (with fewest proglottids) proteocephalid tapeworms. Further integrative studies may confirm their close relatedness prompting a new classification scheme for this subgroup.

Historically, fish proteocephalids have been associated with a strict (oioxenous) (sensu Caira et al., 2003) host specificity and a narrow geographical distribution (de Chambrier and Vaucher, 1999; de Chambrier et al., 2017). However, additional sampling and finer species circumscription using both morphological and molecular data have revealed a more relaxed host specificity in some cases (Alves et al., 2017, 2021a). Our results support these findings as *S*. *emarginata* and *C*. *gillesi*, both previously known only from their type hosts and river basins, were found in new pimelodid and cichlid hosts, respectively, as well as in new localities (see Appendix S1a and Results section). Furthermore, our results confirmed the mesostenoxenous specificity (sensu Caira et al., 2003) of *S*. *spinulifera*, a species known from different river basins in South America and one of the most dominant endoparasites infecting large pimelodid catfishes of the genus *Pseudoplatystoma*. The poor resolution of the clade comprising *S*. *spinulifera* and *S*. *lenha* (type‐species) (clade P of de Chambrier et al., 2015) should be investigated in a separate review of *Spasskyellina*, including the transfer of *S*. *mandi* to a new genus [see Alves et al., 2021a].

The phylogenetic position of representatives of two small genera, *Harriscolex* Rego, 1990, and *Cangatiella* Pavanelli and Santos, 1991, reiterates, on the one hand, the highly homoplastic nature of the scolex morphology, and, on the other, the potential usefulness of the reproductive system and strobilar morphology for higher classification (Scholz et al., 2013; Alves et al., 2017, 2021a,b; Arredondo et al., 2017). The three currently recognized species of *Harriscolex* are primarily characterized by a quadrangular scolex with four uniloculate, inverted triangular suckers, each bearing a cone‐shaped projection at the corners of their anterior margins (Gil de Pertierra and Chambrier, 2013). However, it seems that this feature has been evolved independently in *H*. *kaparari* (type‐species) and *H*. *nathaliae* as they do not form a clade in our tree. Conversely, the only two species of *Cangatiella*–*C*. *arandasi* (type species) and *C*. *macdonaghi*, do form a monophyletic group. Among other features, these species share eggs bearing polar projections and a central furrow in the ventral strobilar surface. This peculiar egg morphology can even be used to diagnose the monophyletic assemblage formed by representatives of *Brooksiella*, *Rudolphiella* and *Cangatiella* (clade J of de Chambrier et al., 2015), as well as to differentiate congeneric species (Gil de Pertierra and Chambrier, 2000).

A long‐standing problem in Proteocephalidae taxonomy is the existence of non‐natural, large, catch‐all genera such as *Proteocephalus* Weinland, 1858, *Monticellia* La Rue, 1911, and *Nomimoscolex* Woodland, 1934 (de Chambrier et al., 2004, 2015, 2017). Given that none of the newly sequenced material assigned to these genera clusters in the same clade of their respective type‐species, these taxa should be transferred to other–probably new–genera to better reflect the evolutionary history of the group. Thus, rearrangements at the generic level are expected for: *Pr*. *vazzolerae*, *Pr*. cf. *hobergi*, *Pr*. *microscopicus*, *Pr*. *sophiae*, *M*. *magna*, *M*. *ventrei*, *N*. *sudobim* and *N*. *admonticellia*. Within these taxa, the sister relationship and ecological similarities between *M*. *ventrei* and *N*. *admonticellia* should be better investigated so that putative morphological synapomorphies can be revealed.

### Phylogenetic relationships in the Onchoproteocephalidea I

Merging the former Proteocephalidea with a subset of elasmobranch‐hosted cestodes within the order Onchoproteocephalidea has been controversial and a matter of intense debate (Arredondo et al., 2014; Alves et al., 2015; Scholz and Kuchta, 2022), yet the monophyly of the family Proteocephalidae has never been challenged (Caira et al., 2014; Caira and Jensen, 2014; de Chambrier et al., 2015, 2017). Our phylogenetic tree did not support the monophyly of the Proteocephalidae, because members of the Acanthotaeniinae and Gangesiinae exhibited a sister relationship with the elasmobranch‐hosted onchoproteocephalids used as an outgroup. The use of a large outgroup sampling allowed us to test the monophyly of the ingroup more rigorously (Grant, 2019), and further studies may use a similar approach to better explore the interrelations between these groups.

The overall topology of the parsimony phylogeny based on the concatenated RNA28S + MT‐CO1 dataset is concordant with the most comprehensive analyses for the proteocephalids (de Chambrier et al., 2015), which was based on the RNA28S gene alone and used model‐based phylogenetic reconstructions. Our results reinforced the artificial nature of the subfamilial classification, yet the early diverging Acanthotaeniinae and Gangesiinae are the most stable groups that can be morphologically characterized. In fact, these two are the only morphology‐based subfamilies with more than one genus that persist in recent taxonomic accounts (de Chambrier et al., 2020; Marick et al., 2023; de Chambrier and Scholz, 2024).

Adding nearly 100 new RNA28S and MT‐CO1 sequences to the proteocephalid phylogeny (a taxon‐wise strategy) did not significantly improve the resolution nor support of the interrelationships among Neotropical proteocephalids, that could allow the recognition of natural groups sharing either morphological, biological or ecological traits. It is hypothesized that the poor resolution and support observed in the recent phylogenies reflect a rapid radiation of species, also mirroring the complex Neotropical fish diversification (de Chambrier et al., 2004, 2015). Even though hard polytomies cannot be ruled out in the evolutionary history of proteocephalids, phylogenies using larger matrices (e.g. based on complete mitochondrial genomes and multiple orthologous nuclear genes) should be developed.

The diversification of fish proteocephalids in the Neotropics remains largely obscured, yet our findings suggest that non‐siluriform fishes played a key role in the initial colonization of this zoogeographical realm by proteocephalids. Later, a notable diversification occurred in catfishes, primarily among pimelodids. The newly sequenced *Pr*. *vazzolerae* (PD072, PS073, PS074) from anostomid fish (Anostomidae), along with *C*. *gillesi* (PD419, PD420) and *Sciadocephalus megalodiscus* Diesing, 1850, from cichlid fishes (Cichlidae), represent some of the earliest diverging Neotropical lineages. The role of the Redtail catfish *Ph*. *hemioliopterus* also is worth mentioning. This relictual catfish, the only living species of the genus *Phractocephalus*, harbours three early diverging Neotropical proteocephalids, namely *S*. *emarginata* (PS036–38, PS102), *Zygobothrium megacephalum* Diesing, 1850 (PD001–03) and *Pr*. *hemioliopteri* de Chambrier and Vaucher, 1997 (PD138) (Alves et al., 2018).

### The high predictive power of machine learning

The ongoing revolution in artificial intelligence, particularly in ML algorithms, has boosted scientific knowledge across diverse fields such as ecology, chemistry, robotics, economics and physics (Jordan and Mitchell, 2015). The popularity of ML approaches has steadily increased in recent years, placing them as an alternative to traditional probability‐based statistical models for data analysis and prediction (Pichler and Hartig, 2023). The wide range of applications within the ML framework (e.g. unsupervised and supervised strategies, deep learning) provides scientists with powerful tools to address complex questions, including those related to ecological, taxonomic and evolutionary challenges (Pichler and Hartig, 2023).

Despite the potential of ML in answering a myriad of questions, just a few studies have employed ML strategies using nonmodel parasitic organisms that are not of medical or veterinary importance (Dallas et al., 2017; Borba et al., 2021; Cruz‐Laufer et al., 2022). Although they tackle unrelated issues, these studies have in common the use of supervised ML algorithms to test the predictive classification power of morphological, morphometrical and life‐history traits. Dallas et al. (2017) found that the parasite community structure is likely to be more important than host and geographical attributes to predict the host range of parasitic helminths, and Borba et al. (2021) showed that egg morphology and morphometrics, along with ecological and geographical metadata may be useful to predict capillariid nematode species accurately. Conversely, Cruz‐Laufer et al. (2022) found only a moderate model performance using morphometrics to predict clade affiliation of ectoparasitic flatworms in cichlid fishes across Africa. To the best of our knowledge, our study is the first to apply supervised ML to test whether host and biogeographical attributes of nonmodel parasitic organisms can accurately predict clade affiliation, potentially informing evolutionary relationships in a family‐level framework.

Host–parasite associations are intrinsically linked to interrelated factors such as geographical distribution, natural history and evolutionary relationships (Poulin, 2014; Penczykowski et al., 2016; Dallas and Becker, 2021). Altogether, these factors structure the parasite communities and drive the diversification of these organisms. Therefore, phylogenetic relationships of parasitic taxa are deeply influenced by host and biogeographical constraints such that phylogenetically closely related hosts in a given zoogeographical realm tend to have phylogenetically closely related parasites (Dallas et al., 2017; Dallas and Becker, 2021). Although host‐switching events are common among parasites, they are more frequent in a reduced spatial scale and usually constrained by the host phylogeny (D'Bastiani et al., 2023).

Our study suggests that whereas traditional character optimization methods show a poor correlation between host/biogeographical attributes and the proteocephalid tree, supervised ML methods can accurately predict proteocephalid clades in a multidimensional space. Additionally, the clade perturbation test validated the ML analyses as a strong linear relationship between the level of label perturbation and the resulting assignment to clades (i.e. the model's accuracy negatively correlates with the degree of perturbation).

Phylogenetic comparative methods (PCMs) also are broadly applied to evaluate the predictive power of traits such as morphology, morphometrics, natural history and ecology in the context of phylogenetic trees within a multidimensional space (Garamszegi, 2014; Revell and Harmon, 2022). The reason for not applying these methods in the present study is that all PCM methods rely on evolutionary models (additional *ad hoc* assumptions required) and incorporate phylogenetic trees into the analyses. Our approach here was to use external (to the tree), phylogeny‐free evidence to correlate host and biogeographical metadata with the evolutionary history of the proteocephalids. This approach informed us that the generated cladogram is biologically informative. Moreover, unlike PCM methods, our approach can handle hierarchic features otherwise inadequate for homology hypotheses and allows the use of different qualitative data in the same analysis.

The Random Forest algorithm used herein allowed us to discriminate the most important attributes investigated, but it is not our goal to spot a single most‐informative feature. Instead, we aimed to combine multiple layers of evidence that might correlate with the intricate and complex evolution of proteocephalids. In this context, highly important features without perturbation, such as “Pimelodidae,” “South America” and “Siluriformes” (see Fig. 9a), may reflect oversampled features. Nevertheless, it is clear that less biologically relevant attributes gain importance at high clade perturbation levels (see Fig. 9b,c).

We acknowledge that our understanding of the proteocephalid evolution is far from being completely elucidaded, but applying the powerful toolkit offered by ML methods provides important avenues to tackle this complex subject.

## Conflict of interest

None declared.

## Supporting information


**Appendix S1.** List of supplementary tables (both in csv and xlsx formats).
**Appendix S1a.** List of newly collected tapeworms and associated metadata.
**Appendix S1b.** Summary data of all tapeworm sequences used in the phylogenetic analysis.
**Appendix S1c.** Codification of characters and associate states for the character optimization analysis.
**Appendix S1d.** States of character for each terminal used in the character optimization analysis.
**Appendix S1e.** Categorial attributes and associated states used in the random forest analysis.
**Appendix S1f.** Selected clades used for training the Random Forest algorithm.
**Appendix S1g.** List of synonyms used through the analyses.
**Appendix S2.** Individual character optimization files.
**Appendix S2a.** List of non‐ambiguous transformations and their type.
**Appendix S2b.** Detailed character matrix used to generate the TNT file.
**Appendix S2c.** Legend for characters and character states.
**Appendix S2d.** Non‐ambiguous synapomorphies of each node.
**Appendix S2e.** Character matrix in TNT file.
**Appendix S2f.** Tree in Newick format containing branch labels (nodes).
**Appendix S2g.** PDF file of the tree including transformations in nodes with three or more terminals.
**Appendix S3.** Alignment and Tree search files.
**Appendix S3a.** Alignment‐ready for the tree search in TNT (28S rRNA).
**Appendix S3b.** Alignment‐ready for the tree search in TNT (MT‐CO1).
**Appendix S3c.** Alignment‐ready for the tree search in TNT (TOTAL).
**Appendix S3d.** TNT script for the tree search.
**Appendix S3e.** Most parsimonious trees in Newick format.
**Appendix S3f.** TNT script for summarizing the strict consensus tree.
**Appendix S3g.** TNT script for the branch length assessment.
**Appendix S3h.** TNT script for the branch support and frequency analysis.
**Appendix S3i.** Consensus tree in Newick format (no branch lengths or support values).
**Appendix S3j.** Complete consensus tree (including branch lengths, support and frequency values).
**Appendix S3k.** Consensus tree, including Goodman‐Bremer and Jackknife values, in PDF format.
**Appendix S4.** Machine learning files.
**Appendix S4a.** Python3 script for the random forest classifier algorithm, including the perturbation test.
**Appendix S4b.** Raw input dataset.
**Appendix S4c.** Curated input dataset.
**Appendix S4d.** Target clades for prediction.
**Appendix S4e.** Accuracy of the random forest model across ten replication runs, ordered by increasing levels of perturbation.
**Appendix S4f.** List of most important features across 10 replication runs, ordered by increasing levels of perturbation.
**Appendix S5.** Maximum likelihood files and associated data.
**Appendix S5a.** Alignment‐ready for the tree search in IQ‐TREE2 (28S rRNA).
**Appendix S5b.** Alignment‐ready for the tree search in IQ‐TREE2 (MT‐CO1).
**Appendix S5c.** Partitions for analyzing the gene alignments simultaneously in IQ‐TREE2 (concatenated RNA28S + MT‐CO1).
**Appendix S5d.** Best evolutionary models, per partition, as chosen by ModelFinder in IQ‐TREE2.
**Appendix S5e.** Script for the tree search in IQ‐TREE2.
**Appendix S5f.** Script for the branch support assessment in IQ‐TREE2.
**Appendix S5g.** Best‐scoring maximum likelihood (ML) tree in IQ‐TREE2, including branch support.
**Appendix S5h.** Maximum likelihood tree used in the sensitivity test within YBIRÁ.
**Appendix S5i.** Parsimony tree used in the sensitivity test within YBIRÁ.
**Appendix S5j.** Arguments used to run the sensitivity test within YBIRÁ.
**Appendix S5k.** Tree with clades match/mismatch between the maximum likelihood and parsimony reconstructions.
**Appendix S5l.** Maximum likelihood tree with selected clades highlighted by colors.
**Appendix S5m.** List of selected clades sorted by color.
**Appendix S5n.** Input data used to run the machine learning analysis. It includes the target clades.
**Appendix S5o.** Input data used to run the machine learning analysis. It includes the host and biogeographical attributes.
**Appendix S5p.** Output data generated in the machine learning analysis. It includes the model's accuracy per replicate and perturbation level.
**Appendix S5q.** Output data generated in the machine learning analysis. It includes the list of attributes sorted by importance.
**Appendix S5r.** Output data generated in the machine learning analysis. It includes the perturbation thresholds.
**Appendix S5s.** Output data generated in the machine learning analysis. It includes a graph with the impact of clade perturbation on the random forest model performance.
**Appendix S5t.** Comparison between the accuracy levels of the machine learning analyses using both the maximum likelihood‐ and parsimony‐based clades.

## Data Availability

Hologenophores were deposited in the Helminthological Collection of the Biosciences Institute (CHIBB), UNESP, Botucatu, São Paulo State (acc. nos CHIBB 796L–845L and 10630, 10631). Newly generated sequences were deposited in the GenBank database (acc. nos RNA28S PQ343932–PQ343989, MT‐CO1 PQ340938–PQ340969 and PQ350129).
