# Supplementary material for: Machine learning models accurately predict clades of proteocephalidean tapeworms (Onchoproteocephalidea) based on host and biogeographical data
Source: Cladistics. 2025 Mar 6;41(3):264–82. doi: 10.1111/cla.12610 (PMC12065121; doi:10.1111/cla.12610)

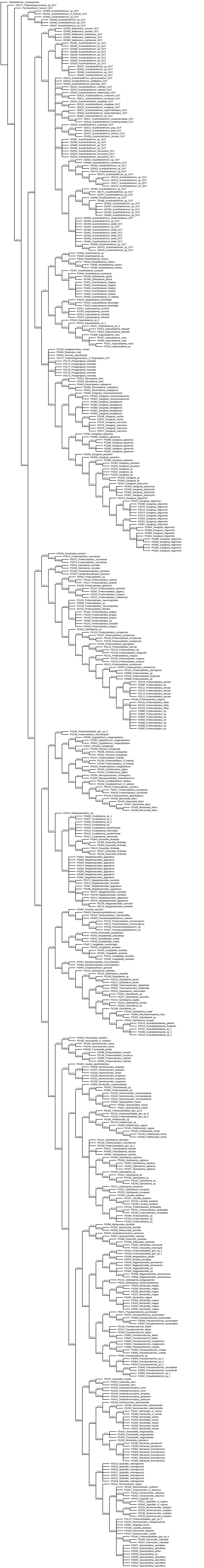

Supplement: Supplementary file 1 — Appendix S1. List of supplementary tables (both in csv and xlsx formats). Appendix S1a. List of newly collected tapeworms and associated metadata. Appendix S1b. Summary data of all tapeworm sequences used in the phylogenetic analysis. Appendix S1c. Codification of characters and associate states for the character optimization analysis. Appendix S1d. States of character for each terminal used in the character optimization analysis. Appendix S1e. Categorial attributes and associated states used in the random forest analysis. Appendix S1f. Selected clades used for training the Random Forest algorithm. Appendix S1g. List of synonyms used through the analyses. Appendix S2. Individual character optimization files. Appendix S2a. List of non‐ambiguous transformations and their type. Appendix S2b. Detailed character matrix used to generate the TNT file. Appendix S2c. Legend for characters and character states. Appendix S2d. Non‐ambiguous synapomorphies of each node. Appendix S2e. Character matrix in TNT file. Appendix S2f. Tree in Newick format containing branch labels (nodes). Appendix S2g. PDF file of the tree including transformations in nodes with three or more terminals. Appendix S3. Alignment and Tree search files. Appendix S3a. Alignment‐ready for the tree search in TNT (28S rRNA). Appendix S3b. Alignment‐ready for the tree search in TNT (MT‐CO1). Appendix S3c. Alignment‐ready for the tree search in TNT (TOTAL). Appendix S3d. TNT script for the tree search. Appendix S3e. Most parsimonious trees in Newick format. Appendix S3f. TNT script for summarizing the strict consensus tree. Appendix S3g. TNT script for the branch length assessment. Appendix S3h. TNT script for the branch support and frequency analysis. Appendix S3i. Consensus tree in Newick format (no branch lengths or support values). Appendix S3j. Complete consensus tree (including branch lengths, support and frequency values). Appendix S3k. Consensus tree, including Goodman‐Bremer and Jackknife values, in PDF [file cla12610-sup-0042-AppendixS5k.pdf › cla12610-sup-0042-AppendixS5k.pdf]
